# Electrodeposition of Gold Nanostructures at the Interface of a Pickering Emulsion

**DOI:** 10.1002/celc.201800398

**Published:** 2018-05-17

**Authors:** Samuel G. Booth, Rafgah G. Alghamdi, Domagoj Belić, Mathias Brust

**Affiliations:** ^1^ Department of Chemistry University of Liverpool Liverpool L69 7ZD UK; ^2^ Division of Materials Physics Ruđer Bošković Institute Bijenička C.54 10000 Zagreb Croatia

**Keywords:** cryo-TEM, deposition, electrochemistry, electron transfer, Pickering emulsions

## Abstract

The controlled electrodeposition of nanoparticles at the surface of an emulsion droplet offers enticing possibilities in regards to the formation of intricate structures or fine control over the locus or duration of nanoparticle growth. In this work we develop electrochemical control over the spontaneous reduction of aqueous phase Au(III) by heterogeneous electron transfer from decamethylferrocene present in an emulsion droplet – resulting in the growth of nanoparticles. As gold is a highly effective conduit for the passage of electrical current, even on the nanoscale, the deposition significantly enhances the current response for the single electron transfer of decamethylferrocene when acting as a redox indicator. The nanostructures formed at the surface of the emulsion droplets were imaged by cryo‐TEM, providing an insight into the types of structures that may form when stabilised by the interface alone, and how the structures are able to conduct electrons.

Liquid/liquid interfaces have numerous applications in phase separation procedures, sensing, catalysis, and growth or assembly of nanoscale structures. Due to the high surface tension between two immiscible liquids there is a significant energetic benefit to the assembly of nanostructures between the two fluids to reduce the area in contact. Such systems have been observed since early work on the spontaneous deposition of metals or the development of Pickering emulsions.^1^ The thermodynamic factors have been explained in the works of Binks et al., with informative graphical depictions of the energy well provided for voltage induced assembly by Flatte et al. and for spontaneous particle assembly in the recent work of Smirnov and co‐workers.^2^ The adsorption of pre‐formed structures and in situ metal deposition has been covered in a number of comprehensive review articles.2d,^3^ Electrochemical control over the deposition of metallic structures at a liquid/liquid interface has been an area of high interest following the development of a 4‐electrode system which enables researchers to apply a potential directly to the interface.^4^ In such a system control over the reduction of a metal precursor may be provided by an overpotential at the liquid/liquid interface to drive interfacial electron transfer or alternatively through the Gibbs free energy of ion transfer $\Delta G_{org}^{aq}$ between the two phases.^5^

However, it is also possible to control heterogeneous redox reactions at the surface of a Pickering emulsion, including metal reduction protocols.^6^ Sachdev et al. have shown that an organic phase reducing agent is able to drive the deposition of gold at the surface of well‐defined droplets within a microfluidic system.^7^ This system interested us in relation to the work of Mirceski and co‐workers on the deposition of gold and silver at the surface of a thin film electrode.^8^ Such work showed that the spontaneous or controlled metal deposition blocked the interface to ion transfer and therefore reduced the current response observed during cyclic voltammetry (CV).^9^ In contrast, the 3‐phase boundary with a Pickering emulsion has shown that nanoparticles have the capacity to transfer electrons from the electrode surface through an electron hopping mechanism.^10^ In this work we report on the electrochemically controlled deposition of gold nanoparticles at an aqueous/trifluorotoluene (TFT) interface. The growth has been examined through electrochemistry, with the use of cryo‐TEM to image the structure of the interfacial deposits. The electrochemically controlled deposition allows for the regeneration of the reducing agent therefore establishing the possible development of a system for continuous colloidal gold formation.

As shown previously, ferrocene and its derivatives are effective reducing agents for aqueous [AuCl_4_]^−^.^7^, ^8^,^11^ In this case decamethylferrocene (DMFc) was utilised as it is stable to photo‐degradation and has a very low solubility in the aqueous phase (Figure S1). Scheme 1 depicts the processes occurring within an emulsion microdroplet on the electrode surface. Initially there is a spontaneous electron transfer reaction between the DMFc within the TFT droplet and aqueous phase [AuCl_4_]^−^ (1). If a low DMFc concentration is employed, then the species is rapidly depleted,forming DMFc^+^ within the droplet as the gold is deposited. Under an applied potential, DMFc is instead replenished at the electrode enabling further gold deposition (2). Once the Au^0^ begins to build up at the surface of the droplet it is able to act as a conduit for the electrons between the electrode and the reducing agent (3). As this process increases the effective surface area of the electrode the current for the $DMFc\;\vec{\leftarrow }{DMFc}^{+}$ redox reaction is seen to increase significantly.

**Scheme 1:**
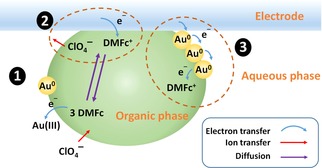
Illustration of the reactions occurring within the emulsion droplet. (1) Organic phase DMFc reduces aqueous Au(III) [AuCl_4_]^−^, forming nanoparticles. The charge is compensated by transfer of background electrolyte. (2) DMFc^+^ is regenerated into DMFc at the electrode surface. (3) As Au^0^ grows on the surface of the droplet DMFc^+^ can be reduced through electron hopping.

Equation 1 gives the overall reaction for the reduction of Au(III) by DMFc, where *aq*, *org* and *i* refer to the aqueous phase, the TFT phase and the interface respectively.

(1) $${H^{+}{\left[{AuCl}_{4}\right]}^{-}}_{aq}+{3DMFc}_{org}\to {{Au}^{0}}_{i}+{{3DMFc}^{+}}_{org}+{3\;{Cl}^{-}}_{aq}+{HCl}_{aq}$$

The reduction leads to the build‐up of negative ions within the aqueous phase and positive ions within the organic phase. The electroneutrality of the interface is maintained during the reduction procedure through the transfer of background electrolyte, ClO_4_^−^ ions, between the phases (Equation (2).

(2) $${{3\left[ClO_{4}\right]}^{-}}_{aq}\to {{3\left[ClO_{4}\right]}^{-}}_{org}$$

The system can be evaluated through cyclic voltammetry. In order to see the initial depletion of the organic phase DMFc through the spontaneous reduction of aqueous [AuCl_4_]^−^ an emulsion was formed and allowed to stand for 10 minutes. Subsequently, the CV was conducted with an onset potential above that required to reduce DMFc^+^. The E_1/2_ for the reversible single electron transfer of DMFc occurs at ca −0.005 V vs Ag/AgCl. When the CV was performed with an initial potential of 0.0 V it shows that on the forward scan there was no signal for the oxidation of DMFc as the species has undergone complete conversion. On reversing the scan direction, the peak corresponding to the reduction of DMFc^+^ is clearly present. Subsequent scans were conducted, again with a 10 minute wait time (open circuit potential) between measurements. The inset of Figure 1 shows the 2^nd^, 3^rd^ and 4^th^ scan. On each scan the positive peak current increases. As [AuCl_4_]^−^ is depleted in the aqueous phase, less DMFc is consumed through reduction leading to a more symmetrical CV.

**Figure 1:**
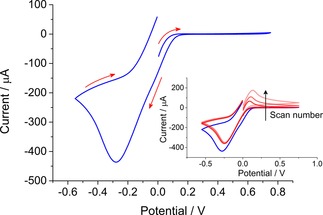
Cyclic voltammogram for emulsion droplets formed when 250 μL of TFT containing 20 mM DMFc and 0.1 M TBAClO_4_ were added to 4 mL of 1.45 mM HAuCl_4_ solution with 0.1 M LiClO_4_. CV scans were performed at 200 mV s^−1^. The inset shows multiple scans, the delay between each scan was 10 minutes.

As discussed with reference to the overall reaction scheme the gold, once present at the droplet interface, should be able to act as a source and sink of electrons enabling transfer between the electrode surface and DMFc^+^ species. This is in marked contrast to a thin film system where there is no direct contact between the electrode and the growing nanostructures. CV analysis makes it possible to see clear differences in the behaviour in the emulsion system compared with that of a thin film (Figure 2). In these systems the samples were assembled and measured without any initial wait time in order to follow the complete reduction process.

**Figure 2:**
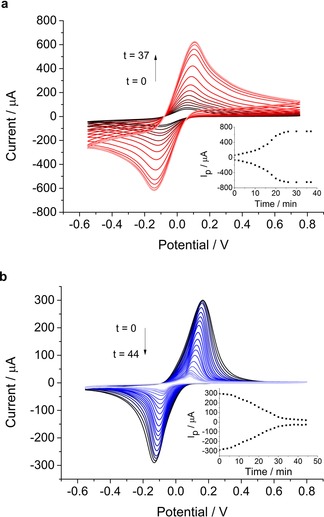
Cyclic voltammetry in (a) emulsion droplets formed by 300 μL of TFT in 6 mL aqueous solution or (b) an organic TFT thin film on the electrode surface. In both cases TFT contained 20 mM DMFc and 0.1 M TBAClO_4_ and the aqueous phase contained 1.1 mM H^+^[AuCl_4_]^−^ and 0.1 M LiClO_4_. CVs were performed at 200 mV s^−1^. The CVs started at −0.6 V, each time plotting the second of 2 scans. The samples were left at open circuit potential between measurements.

In the presence of [AuCl_4_]^−^ in the emulsion system (Figure 2a) the growth of Au nanoparticles results in a dramatic enhancement of the DMFc current response. The figure inset shows that the current rises slowly at first, followed by a more rapid increase before reaching a plateau. This increase in current is due to electron transfer through the Au species to the electrode surface via an electron hopping mechanism, comparable to that proposed by Marken and co‐workers.10a Therefore the growth of gold over the droplet surface, in direct contact with the electrode, causes an increase in the electrode surface area and accordingly, an increase in the current response. This response is significantly different to deposition on a thin organic film (Figure 2b).^8^ When a thin film is formed there is no direct contact between the electrode and the interface between the two phases and therefore as the metal grows it only acts to reduce the organic/aqueous interfacial area.

Therefore, if instead of an emulsion, we form a thin film of the organic phase on the electrode the current decreases dramatically with time until it is almost completely suppressed by blocking of the background electrolyte transfer (CVs and a schematic of the mechanism are shown in Figure S3). In comparison, the response in the absence of [AuCl_4_]^−^ (Figure S4) shows that the signal remains largely unaltered with time. There is a slight decrease in the current which would be linked to the concentration gradient of DMFc^+^ leading to diffusion away from the electrode.^12^ The active surface area of the electrode can be examined by comparing the emulsion droplet and thin film configurations. For a thin film in the absence of gold, the current peak maximum for the oxidation of DMFc (I_p_) is ∼360 μA (data not shown). This value indicates that when the electrode is immersed in the emulsion, the droplets attach to the electrode with a coverage of ca 30 % of the electrode area. Examining the gold growth over time, there is ∼175 % increase in current. This suggests a significant amount of nanoparticle formation creating structures which have a sufficiently short inter‐particle spacing to allow electron tunnelling between particles.

Whilst not under electrochemical control, we have been able to image the particles on the surface of an emulsion in situ by cryo‐TEM (Figure 3 and SI). There have been some recent reports into the use of cryo‐TEM to image particles in emulsion droplets and vesicles as the technique does not require any drying steps and therefore offers a better representation of such systems than traditional EM.^13^ The emulsion was formed using sonication in order to form droplets that were small enough to provide sufficient contrast to image under TEM. As the DMFc species could not be replenished in the absence of electrodes, a higher initial concentration was used (100 mM instead of 20 mM). Figure S6 shows the electrochemical response for this system indicating an enhancement of the current in the presence of [AuCl_4_]^−^, verifying the applicability to the electrochemical system. Microscope images of the droplets under electrochemical conditions are provided for comparison (Figure S7).

**Figure 3:**
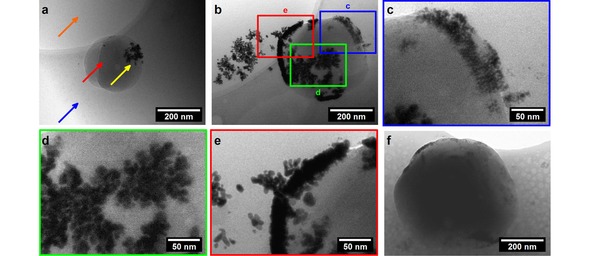
Cryo‐TEM images of a droplet of oil in water emulsion. The aqueous phase contains 0.9 mM HAuCl_4_ and 0.1 M LiClO_4_. The TFT phase contains 100 mM DMFc and 0.1 M TBAClO_4_. The phases were emulsified by sonication prior to the addition of HAuCl_4_. The sample was allowed to react for 5 minutes and then dropped onto a TEM grid for flash freezing. (a) The orange arrow points to a hole in the carbon TEM grid, the blue arrow points to the start of the ice phase indicating that the oil droplet is still contained within a bulk aqueous solution, the red arrow indicates the TFT droplet, and the yellow arrow identifies gold nanoparticles at the emulsion surface. (b) An emulsion droplet showing different types of gold growth found on the droplet surfaces: (c) individual particles, (d) fractal structures, and (e) dense multilayer species. (f) Shows a larger droplet where the density of the organic phase reduces electron penetration, the presence of gold can still be seen around the edges of the droplet.

When imaging, it was found that the oil droplets would preferentially sit on the carbon film itself, adjacent to the holes in the grid. The arrows in Figure 3a indicates the significant features within the micrographs:‐ the holes in the carbon film (orange), the regions where glass‐like ice had formed from the aqueous phase (blue), the oil droplets (red) and the gold nanoparticles (yellow). Some nanoparticles were found closely associated with oil droplets although not directly attached as in Figure 3b. We suggest that this is due to the blotting step to remove excess liquid which may have lifted some particles from the surface of the droplets. No nanoparticles were visible in the regions between emulsion droplets confirming the strong affinity between the particles and the emulsion interface. Figure 3c, d and e show the different types of particle visible on the surface. Primarily individual or partially aggregated spherical particles (c), fractal growths (d), and dense multilayer regions (e). The fractal growths suggest diffusion limited growth as the [AuCl_4_]^−^ is consumed from the aqueous phase.^14^ We propose that electron transfer to the surface of the electrode would occur readily through regions of dense multilayer structure as identified in (e).

In conclusion, this work demonstrates the possible introduction of electrochemical control to the growth of gold nanoparticles by inhibiting the spontaneous chemical reaction and driving the redox process sequestered within organic droplets attached to an electrode surface. The organic species DMFc acts as both a reducing agent and redox species. A significant increase in current is observed following the deposition of Au which is able to conduct electrons, therefore extending the electrode surface area. If instead there is no direct contact between the 3 phases then the current is reduced through blocking of the interface. The observed increase in electron transfer current is supported by cryo‐TEM which shows extended Au nanostructures on the surface of the droplets. With incisive application of ligands which remove the particles from the surface of the droplets this system may be developed to drive the continuous growth of nanoparticles under electrochemical control.

## **Experimental Section**

To form the emulsion, DMFc (*x* mM DMFc where *x*=20 or 100 mM) and background electrolyte (TBAClO_4_, 0.1 M) in trifluorotoluene (TFT) was added to an aqueous electrolyte (0.1 M LiClO_4_) containing ***y*** mM HAuCl_4_ (*y* was 1.1 mM for 0.3 mL of organic or scaled with volume to keep the same proportions). The phases were shaken for 30 s and the electrodes added. Electrochemical measurements were performed on an Autolab PGSTAT20, using a 3‐electrode set up with a glassy carbon working electrode, coiled Pt counter electrode and Ag/AgCl reference electrode in 1 M KCl. Cryo‐TEM was performed on a FEI Tecnai Spirit G2 BioTWIN TEM using a Gatan 626 cryogenic sample holder at −179 °C. Complete experimental details are provided within the SI.

## Conflict of interest

*The authors declare no conflict of interest*.

## Supporting information

As a service to our authors and readers, this journal provides supporting information supplied by the authors. Such materials are peer reviewed and may be re‐organized for online delivery, but are not copy‐edited or typeset. Technical support issues arising from supporting information (other than missing files) should be addressed to the authors.

Supplementary

## References

[1a] M. Faraday , Philos. Trans. R. Soc. London 1857, 147, 145–181;

[1b] W. Ramsden , Proc. R. Soc. London 1903, 72, 156–164;

[1c] S. U. Pickering , J. Chem. Soc. 1907, 91, 2001–2021.

[2a] B. P. Binks , S. O. Lumsdon , Langmuir 2000, 16, 8622–8631;

[2b] B. P. Binks , J. H. Clint , Langmuir 2002, 18, 1270–1273;

[2c] B. P. Binks, Colloidal Particles at Liquid Interfaces: An Introduction, Cambridge University Press, **2006**;

[2d] M. E. Flatte , A. A. Kornyshev , M. Urbakh , J. Phys. Condens. Matter 2008, 20, 9;

[2e] E. Smirnov , P. Peljo , H. H. Girault , Chem. Commun. 2017, 53, 4108–4111. 10.1039/c6cc09638g 28349148

[3a] R. A. W. Dryfe , Phys. Chem. Chem. Phys. 2006, 8, 1869–1883; 16633673 10.1039/b518018j

[3b] J. B. Edel , A. A. Kornyshev , M. Urbakh , ACS Nano 2013, 7, 9526–9532; 24237248 10.1021/nn405712r

[3c] S. G. Booth , R. A. W. Dryfe , J. Phys. Chem. C 2015, 119, 23295–23309;

[3d] M. D. Scanlon , E. Smirnov , T. J. Stockmann , P. Peljo , Chem. Rev. 2018, 118, 3722–3751. 29381343 10.1021/acs.chemrev.7b00595

[4a] C. Gavach , Mlodnick. T , Guastall. C. R. H. Seances Acad. Sci. Ser. C 1968, 266, 1196–1199;

[4b] J. Koryta , P. Vanysek , M. Brezina , J. Electroanal. Chem. 1976, 67, 263–266;

[4c] P. Vanysek , L. B. Ramirez , J. Chil. Chem. Soc. 2008, 53, 1455–1463.

[5a] Y. F. Cheng , D. J. Schiffrin , J. Chem. Soc. Faraday Trans. 1996, 92, 3865–3871;

[5b] C. Johans , K. Kontturi , D. J. Schiffrin , J. Electroanal. Chem. 2002, 526, 29–35;

[5c] M. Platt , R. A. W. Dryfe , E. P. L. Roberts , Electrochim. Acta 2003, 48, 3037–3046;

[5d] M. Platt , R. A. W. Dryfe , J. Electroanal. Chem. 2007, 599, 323–332;

[5e] A. Uehara , T. Hashimoto , R. A. W. Dryfe , Electrochim. Acta 2014, 118, 26–32;

[5f] S. G. Booth , A. Uehara , S. Y. Chang , J. F. W. Mosselmans , S. L. M. Schroeder , R. A. W. Dryfe , J. Phys. Chem. C 2015, 119, 16785–16792;

[5g] S. G. Booth , S.-Y. Chang , A. Uehara , C. La Fontaine , G. Cibin , S. L. M. Schroeder , R. A. W. Dryfe , Electrochim. Acta 2017, 235, 251–261.

[6a] M. Zhang , L. Wei , H. Chen , Z. Du , B. P. Binks , H. Yang , J. Am. Chem. Soc. 2016, 138, 10173–10183; 27429173 10.1021/jacs.6b04265

[6b] Y. Tianyu , W. Lijuan , J. Lingyan , L. Jifen , Z. Xiaoming , T. Min , M. M. J. , C. Ying , W. Yong , G. Sai , Z. Dongyuan , Y. Hengquan , L. Jian , L. G. Q. Max , Angew. Chem. Int. Ed. 2017, 56, 8459–8463.

[7] S. Sachdev , R. Maugi , J. Woolley , C. Kirk , Z. X. Zhou , S. D. R. Christie , M. Platt , Langmuir 2017, 33, 5464–5472. 28514172 10.1021/acs.langmuir.7b00564

[8a] V. Mirceski , R. Gulaboski , J. Phys. Chem. B 2006, 110, 2812–2820; 16471890 10.1021/jp056627r

[8b] B. Sefer , R. Gulaboski , V. Mirceski , J. Solid State Electrochem. 2012, 16, 2373–2381.

[9] S. Komorsky-Lovric , M. Lovric , F. Scholz , J. Electroanal. Chem. 2001, 508, 129–137.

[10a] S. M. MacDonald , P. D. I. Fletcher , Z. G. Cui , M. C. Opallo , J. Y. Chen , F. Marken , Electrochim. Acta 2007, 53, 1175–1181;

[10b] F. Marken , J. D. Watkins , A. M. Collins , Phys. Chem. Chem. Phys. 2011, 13, 10036–10047. 21487620 10.1039/c1cp20375d

[11] R. Ciganda , J. Irigoyen , D. Gregurec , R. Hernández , S. Moya , C. Wang , J. Ruiz , D. Astruc , Inorg. Chem. 2016, 55, 6361–6363. 27333944 10.1021/acs.inorgchem.6b01183

[12] F. Scholz , S. Komorsky-Lovric , M. Lovric , Electrochem. Commun. 2000, 2, 112–118.

[13a] S. Hamzehlou , M. Aguirre , J. R. Leiza , J. M. Asua , Macromol. 2017, 50, 7190–7201;

[13b] M. P. Grzelczak , A. P. Hill , D. Belic , D. F. Bradley , C. Kunstmann-Olsen , M. Brust , Faraday Discuss. 2016, 191, 495–510. 27420179 10.1039/c6fd00037a

[14] H. Brune , C. Romainczyk , H. Roder , K. Kern , Nature 1994, 369, 469–471.

