# Supplementary material for: Electrodeposition of Gold Nanostructures at the Interface of a Pickering Emulsion
Source: ChemElectroChem. 2018 May 17;5(15):2055–8. doi: 10.1002/celc.201800398 (PMC6099388; doi:10.1002/celc.201800398)
Supplement: Supplementary file 1 — Supplementary [file CELC-5-2055-s001.pdf]

Supporting Information

© Copyright Wiley-VCH Verlag GmbH & Co. KGaA, 69451 Weinheim, 2018

Electrodeposition of Gold Nanostructures at the Interface of a Pickering Emulsion

Samuel G. Booth,* Rafgah G. Alghamdi, Domagoj Belić, and Mathias Brust*© 2018 The Authors. Published by Wiley-VCH Verlag GmbH & Co. KGaA.
This is an open access article under the terms of the Creative Commons Attribution License, which permits use, distribution and reproduction in any medium, provided the original work is properly cited.

Author Contributions

S.B. Conceptualization:Equal; Data curation:Lead; Formal analysis:Equal; Investigation:Supporting; Methodology:-Supporting; Supervision:Supporting; Writing – original draft:Lead; Writing – review & editing:Lead
R.A. Formal analysis:Supporting; Investigation:Lead; Writing – review & editing:Supporting
D.B. Formal analysis:Supporting; Investigation:Supporting; Methodology:Lead; Writing – review & editing:Supporting
M.B. Conceptualization:Equal; Funding acquisition:Lead; Methodology:Equal; Project administration:Lead; Resources:Lead; Supervision:Lead; Validation:Lead; Writing – review & editing:Supporting

Experimental

Chemicals

Gold(III) chloride ($HAuCl_4$, 99.995%), lithium perchlorate ($LiCLO_4$, 99.99%), decamethylferrocene (DMFc, ≥95%), α,α,α-trifluorotoluene (TFT, ≥99%) and tetrabutylammonium perchlorate ($TBAClO_4$, ≥99%) were all purchased from Sigma-Aldrich (Dorset, UK) and used without further purification. Ultra-pure water (Mili-Q, 18.2 MΩ) was used for all solutions.

In situ growth of Au nanoparticles to form Pickering emulsions

In this system the redox mediator also acts to reduce $[AuCl_4]^-$. ***x*** mM DMFc is present within the organic phase (where ***x*** = 20 or 100 mM) along with background electrolyte ($TBAClO_4$, 0.1 M). The volume of the organic solution was between 0.05 and 0.5 mL, and is 0.3 mL unless otherwise stated. The organic phase was added to an aqueous solution (4 or 6 mL) which contained ***y*** mM $HAuCl_4$ (***y*** was 1.1 mM for 0.3 mL of organic or scaled to keep the same proportions) and background electrolyte (0.1 M $LiClO_4$). The two phases were shaken for 30 seconds. The electrodes were then directly immersed in the emulsion solution.

Electrochemical Measurements

A standard 3-electrode set up was used for all of the measurements. The working electrode was glassy carbon (diameter 3 mm), the counter electrode was a coiled Pt wire (99.99%) and the reference electrode was Ag/AgCl in a 1 M KCl solution connected through a glass frit. Cyclic voltammetry was performed on an Autolab PGSTAT20 potentiostat (Metrohm, Runcorn, UK).

Cryo-TEM

For TEM, nano-emulsion samples were prepared by sonicating a 50 µL solution of TFT (100 mM DMFc and 0.1 M $TBAClO_4$) in 6 mL of 0.1 M $LiClO_4$ for 5 minutes. After sonication a concentrated gold solution (50 mM, 0.11 mL) was added to the emulsion mixture and left for 5 minutes before dropping 5 µL onto a TEM grid. The grids used were holey carbon film on 300 mesh copper (Agar Scientific). The grid was placed in a FEI Vitrobot Mk2 vitrification system set to 8 °C and a relative humidity of 98%. The samples were blotted twice (for 2 s) and plunged into liquid ethane before transferring to a Gatan 626 cryogenic sample holder where a temperature of -179 °C was maintained during imaging. TEM imaging was performed on a FEI Tecnai Spirit G2 BioTWIN TEM operating at an accelerating voltage of 120 kV, using an Olympus-SIS MegaView III digital camera. Cryo-TEM was performed at a low electron dose (6.8 e^- $Å^{-2}s^{-1}$, total dose per image < 100 e^- $Å^{-2}$).

Cryo-SEM

For cryo-SEM images the emulsion was prepared using the same method as for cryo-TEM. The emulsion was then dropcast in liquid nitrogen. The frozen droplet was placed on an SEM stub for imaging using an FEI Quanta 250 FEG environmental scanning electron microscope. The microscope is equipped with a Peltier stage which was maintained at -15 °C, the chamber was set at 95% relative humidity. In these conditions the organic phase evaporates quickly whilst the aqueous phase (ice) is maintained for longer, allowing for continuous cryo-SEM imaging of the sample.

Cyclic voltammetry of DMFc in an aqueous phase

In order for the reaction to be controlled electrochemically it is important that the redox species is insoluble in the aqueous phase and that there is therefore no direct reaction between an aqueous phase species and the electrode surface. This was examined through 2 blank experiments. In (1) an aqueous electrolyte solution was saturated with DMFc to see if the concentration dissolved was detectable in the CV. Whilst in (2) DMFc was present in a TFT droplet within the aqueous phase, however, with no direct contact between the TFT phase and the working electrode. The CVs from both experiments (Figure S1) show no clear electron transfer peaks therefore proving that the reaction being detected in the other systems is the organic phase electron transfer between DMFc and $DMFc^+$.

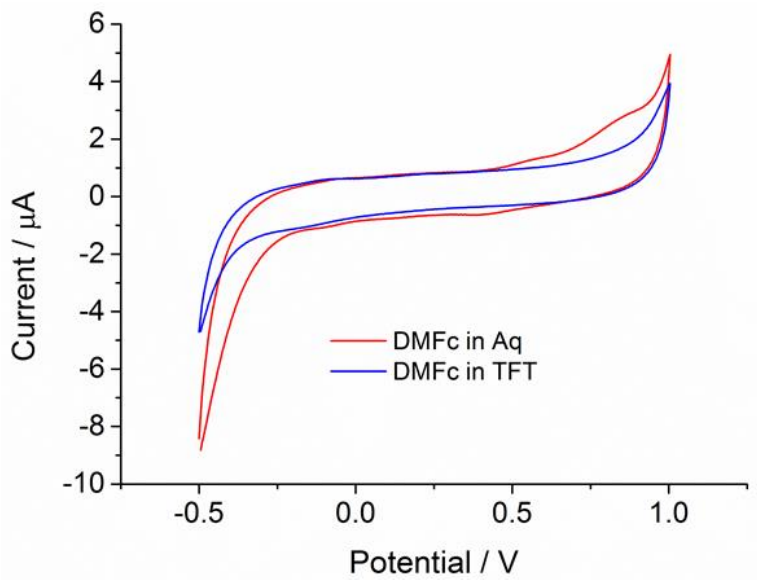

Figure S1. Cyclic voltammetry for DMFc showing the low solubility in aqueous solutions. The red scan corresponds to an aqueous solution (0.1 M $LiClO_4$) saturated with DMFc. The blue scan shows the response from the aqueous solution (0.1 M $LiClO_4$) when a TFT droplet (300 µL, 0.1 M $TBAClO_4$) containing 20 mM DMFc is present but not in contact with the electrode.

Cyclic voltammetry for aqueous [AuCl$_4$]$^-$

In the absence of the organic phase reducing agent, DMFc, it is still possible for some Au deposition to occur. Instead of deposition on the surface of the droplet deposition in this case occurs on the surface of the electrode. This may occur in the presence or absence of the TFT phase (Figure S2). There is some variability in the response due to the deposition of Au on the carbon surface. This is prevalent within the literature and was addressed in the recent work of Lomax et al.[1]

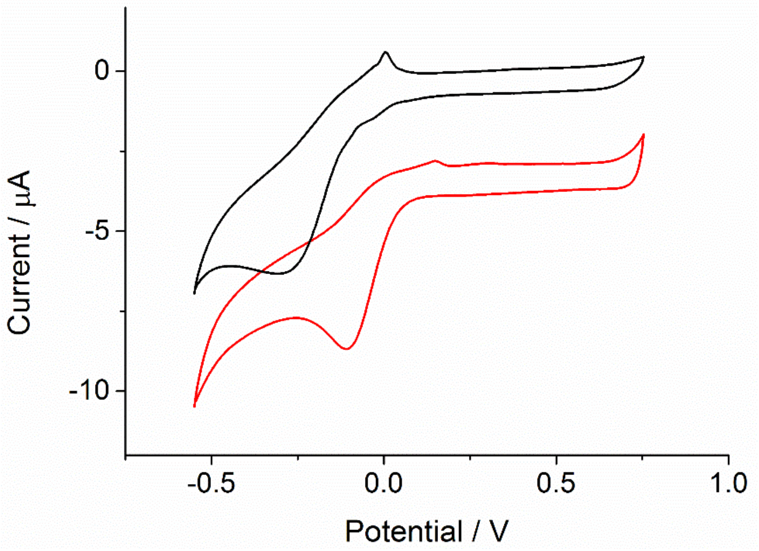

Figure S2. Cyclic voltammetry for the aqueous phase [AuCl$_4$]$^-$ (1 mM) in the presence (red) and absence (black) of organic TFT droplets. Scan rate of 50 mV s^{-1}.

Cyclic voltammetry for the addition of [AuCl$_4$]$^-$ in a thin film configuration

In order to verify the proposed mechanism, which relies on the deposited gold acting to extend the electrode surface area, the organic phase was deposited as a thin film on the electrode surface ensuring that there was complete coverage of the electrode area. As the gold grows in this system, the signal from DMFc decreases dramatically. This is in accordance with previous results which show that the gold deposit blocks the interface and therefore the transfer of ClO_4^- ions. Charge neutrality cannot be maintained during the growth and this inhibits the single electron transfer process (Figure S3).[2] When compared with the results reported in Figure 2, for deposition on an emulsion droplet, this clearly demonstrates that, when in direct contact with the electrode surface, the gold acts to extend the surface area of the electrode leading to an enhancement of the current.

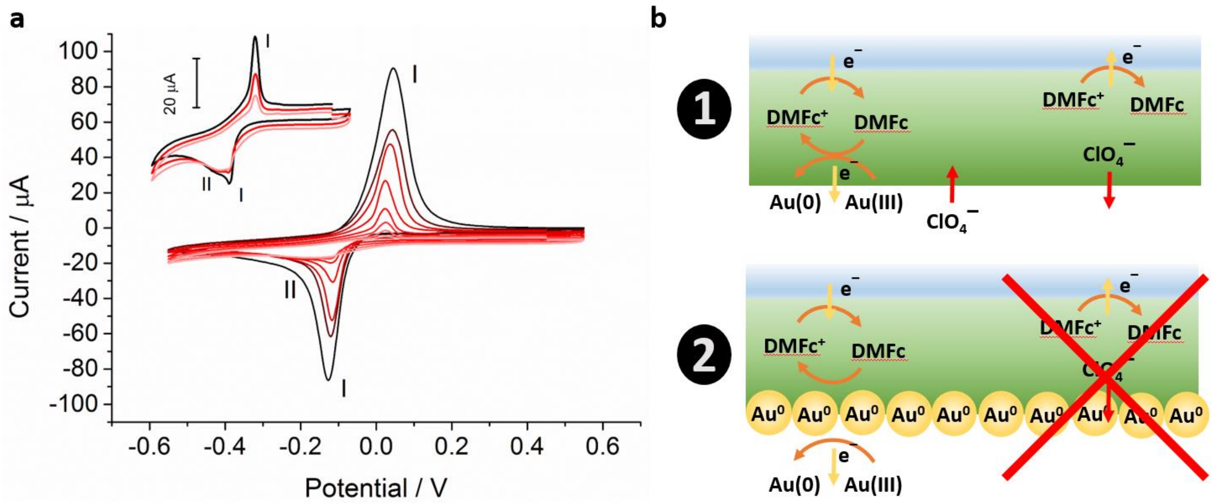

Figure S3. (a) gold growth at a thin film modified electrode. 0.5 µL of TFT containing 20 mM DMFc and 0.1 M $TBAClO_4$ was dropped on the glassy carbon electrode and immersed in a solution of 1.1 mM $HAuCl_4$ and 0.1 M $LiClO_4$. The scan rate was 100 mV s^{-1}. (I) indicates the peaks associated with the DMFc redox couple and (II) indicates irreversible gold deposition on the surface of the thin film. The inset shows the last 3 scans alone featuring a more prominent irreversible peak (II). (b) schematic illustrations of the gold deposition at the beginning of the reaction (1) and as gold growth has occured on the surface of the film (2).

Cyclic voltammetry for a droplet emulsion in the absence of aqueous $[AuCl_4]^-$

If the emulsion is formed in the absence of $[AuCl_4]^-$ the CV shows the clear electrochemical response for DMFc on the electrode surface (Figure S4). This reaction may occur through direct contact between the TFT phase and the electrode. Over time the response is stable, however, with a slight decrease in current. This response is significantly different to that in the presence of $[AuCl_4]^-$ either in the form of an emulsion or as a thin film.

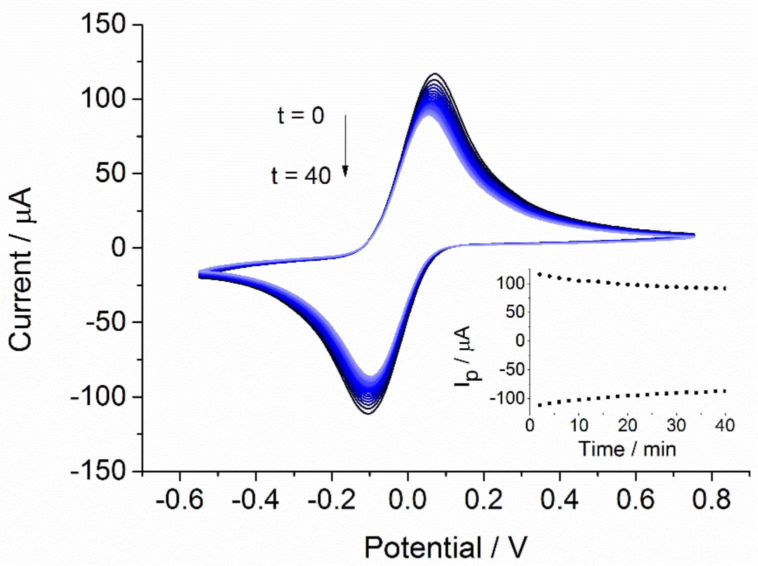

Figure S4. Cyclic voltammetry for an emulsion of 300 µL of TFT containing 20 mM DMFc and 0.1 M $TBAClO_4$ in 6 mL aqueous solution containing 0.1 M $LiClO_4$. The scan rate was 200 mV s^{-1}. The CVs started at -0.6 V, each time plotting the second of 2 scans. The samples were left at open circuit potential between measurements.

Cyclic voltammetry for the addition of $[AuCl_4]^-$ following emulsion formation

It is possible to form the emulsion droplets on the electrode surface before subsequently adding Au(III) to the aqueous phase in order to grow the Au NP film on the interface. This was done in order to verify that the dramatic current increase is due to the gold deposition process and not due to an improvement in droplet stability or side reaction. After performing 30 CV cycles with the DMFc containing TFT phase attached to the electrode surface, an aqueous $[AuCl_4]^-$ solution (50 mM, 132 µL) was injected into the aqueous phase. This triggered the reduction of Au on the surface of the previously formed droplets through heterogeneous electron transfer with DMFc present in the TFT phase. As can be seen (Figure S5) this leads to an increase in the signal response due to the presence of the Au particles which are able to pass the current through electron hopping to the electrode surface therefore increasing the signal from the DMFc present in the organic phase. The enhancement in current response for the addition of gold after the emulsion is formed is always significantly lower than enhancement seen when gold is present prior to the addition of TFT. We suggest that gold formed during the emulsification is able to attach directly to the electrode surface and therefore create a better contact for electron transfer.

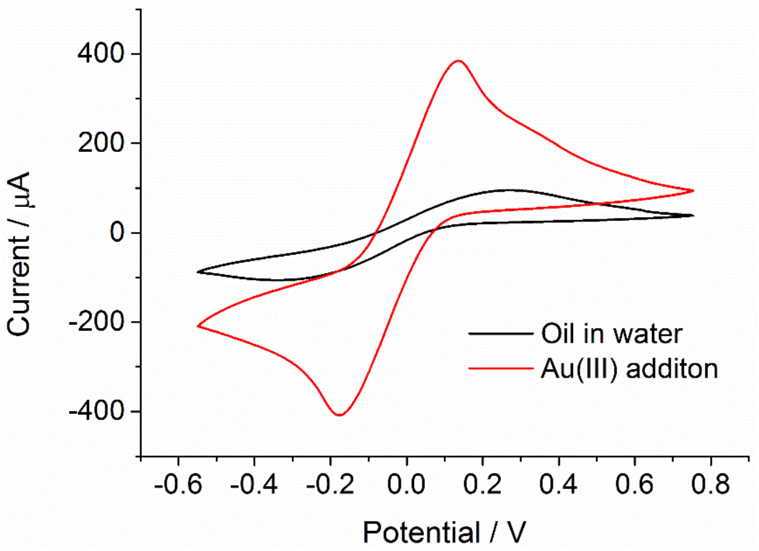

Figure S5. Cyclic voltammetry for TFT in water emulsion, before (black) and after (red) the addition of a concentrated $[AuCl_4]^-$ solution to the aqueous phase. The scan rate was 200 mV s^{-1}, the blank and Au CVs are the 30th cycle in each case.

Cyclic voltammetry of samples prepared under the same conditions as samples for cryo-TEM

As described in the main paper, sonication was used to form emulsion droplets small enough to image in TEM. A higher DMFc concentration (100 mM) was used to reduce more gold without the use of electrochemistry. The corresponding CVs are shown in Figure S6. In the presence of $HAuCl_4$ the DMFc reduction appears to show 2 peaks. We suggest that this is due to the reduction of solution and physisorbed species. In these smaller droplets the relative concentration of physisorbed species is high enough compared to the bulk to show an apparent splitting in the peak.

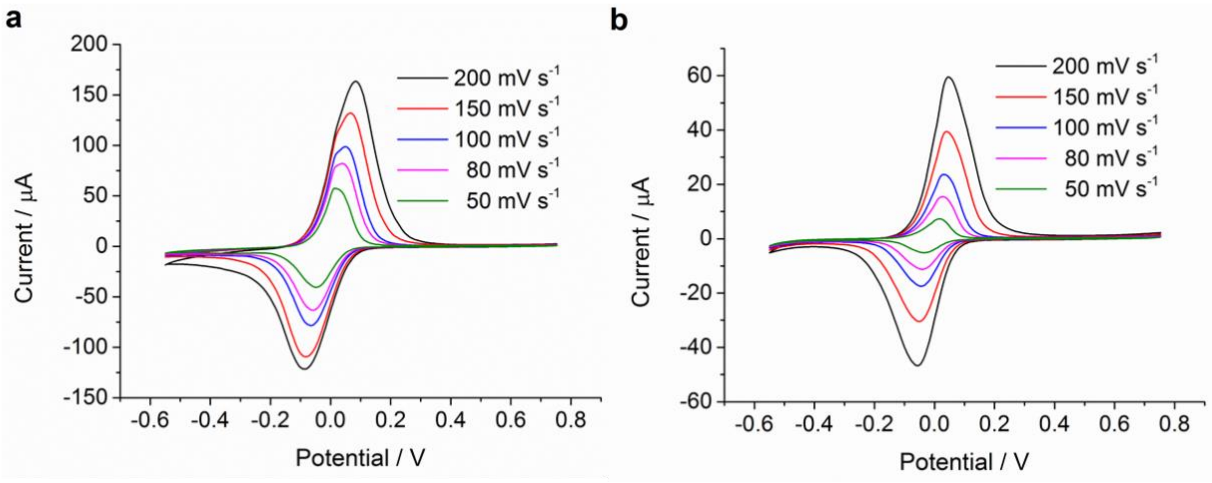

Figure S6. Cyclic voltammetry for TFT in water emulsion matching the conditions used to produce the cryo-TEM samples:- 6 mL aqueous phase (0.1 M $LiClO_4$) and 50 µL TFT (100 mM DMFc and 0.1 M $TBAClO_4$) (a) in the presence (0.9 mM) and (b) absence of $HAuCl_4$ in the aqueous phase.

Optical microscope images of an emulsion showing the influence of gold deposition

In order to show the influence of gold deposition on the formation of an emulsion, microscope images were taken shortly after the emulsion formation. These images compare the same volume of oil in water with different gold and DMFc concentrations (Figure S7). What can be seen is that in the presence of deposited gold species, there is a higher density of small oil droplets. With a higher gold and DMFc concentration, leading to further gold deposition, this effect is further enhanced. A full size distribution was not performed as there will be emulsion droplets that are too small for the resolution of the microscope.

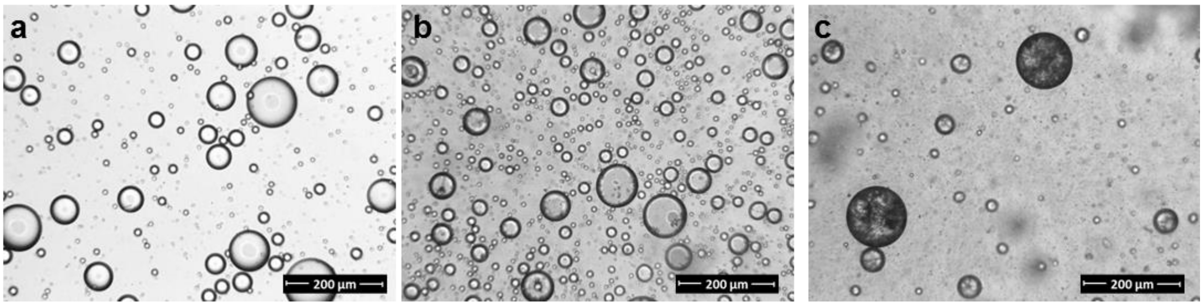

Figure S7. Optical microscopy images of the emulsion formed from TFT (0.3 mL) in water (6 mL). The phases contain background electrolyte (0.1 M $TBAClO_4$ and 0.1 M $LiClO_4$ in TFT and the aqueous phase respectively). (a) bare electrolyte system, (b) 1.1 mM $HAuCl_4$ in the aqueous phase and 20 mM DMFc in the organic phase, and (c) 4.16 mM $HAuCl_4$ in the aqueous phase and 70 mM DMFc in the organic phase. Images were collected 10 minutes after emulsion formation.

Cryo-SEM images of a droplet of oil in water emulsion following gold deposition
An emulsion was formed by sonication following the same process as described for cryo-TEM in the main article. Directly after forming the emulsion, a droplet was collected and pipetted into liquid nitrogen. The sample was then imaged at -15 °C. As the aqueous phase (ice) begins to evaporate, the confined TFT droplets are exposed to the atmosphere. The melting point of TFT is -29 °C so the droplets rapidly evaporate leaving behind the crystallized electrolyte and DMFc species. Figure S8 shows the progression recorded in the SEM over approximately 20 minutes. c-f are successive increases in magnification indicated by the red box in the previous figure in each case. In Figure S8f the blue arrow points to the crystals formed within the droplet, whilst the yellow arrow shows some of the gold nanoparticles which have formed on the droplet surface. All of the images captured showed gold nanoparticles on the outside of the imaged emulsion droplets with none visible within the droplet cores (Figure S8 and S9).

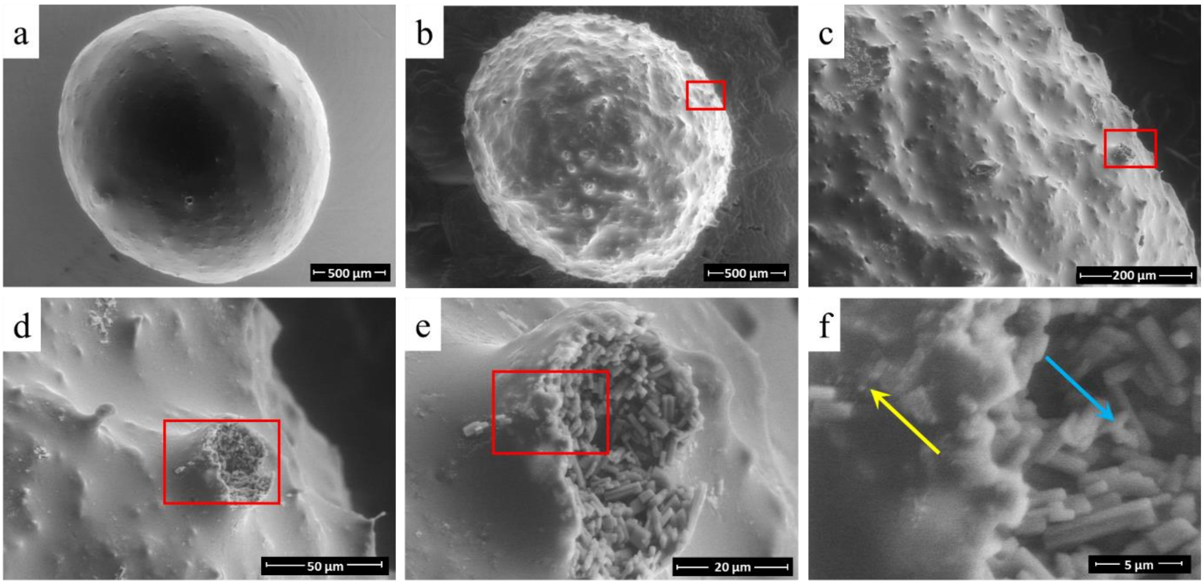

Figure S8. Cryo-SEM images of a droplet of oil in water emulsion. The aqueous phase contains 0.9 mM $HAuCl_4$ and 0.1 M $LiClO_4$. The organic phase (TFT) contains 100 mM DMFc and 0.1 M $TBAClO_4$. The phases were emulsified by sonication prior to the addition of $HAuCl_4$. The emulsion droplet was dropcast in liquid nitrogen before imaging at -15 °C and 95% humidity. (a) the original emulsion droplet, (b) the emulsion droplet after ~20 minutes where the droplet has evaporated slightly and ice has formed on the SEM stub. Images c-f show progressive increases in magnification on an evaporated TFT droplet cavity. In (f) the yellow arrow points to Au nanoparticles and the blue arrow indicates the DMFc and electrolyte crystals formed as the TFT phase is removed.

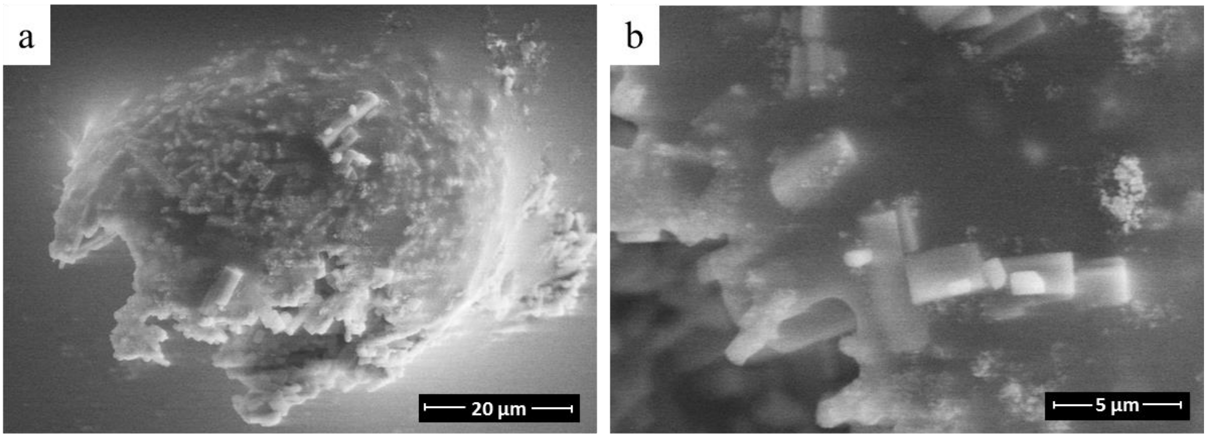

Figure S9. Cryo-SEM images of a droplet of oil in water emulsion. The conditions match those in Figure S8. (a) and (b) show the emulsion droplet at different magnifications following TFT evaporations.

Cryo-TEM of emulsion droplets following gold deposition

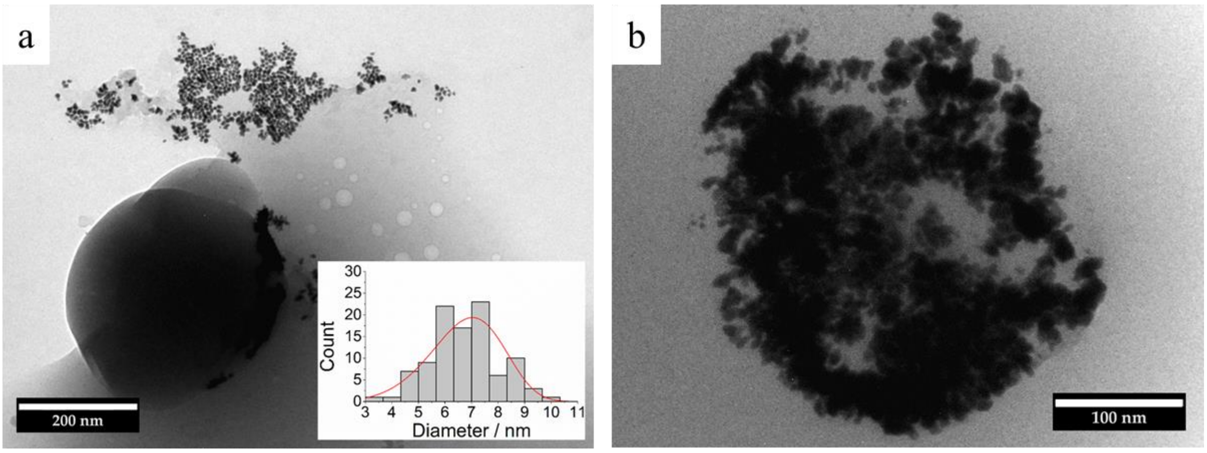

Figure S10. Cryo-TEM images of of a droplet of oil in water emulsion. The aqueous phase contains 0.9 mM $HAuCl_4$ and 0.1 M $LiClO_4$. The organic phase (TFT) contains 100 mM DMFc and 0.1 M $TBAClO_4$. The phases were emulsified by sonication prior to the addition of $HAuCl_4$. The sample was then dropped onto a TEM grid and allowed to react for 5 minutes prior to flash freezing. (a) The gold nanoparticles adjacent to the oil phase were measured with the size distribution included in the inset. (b) An oil emulsion droplet showing a high denisty of Au nanoparticles grown on the droplet surface.

[1] D. J. Lomax, R. A. W. Dryfe, *J. Electroanal. Chem.* **2017**, https://doi.org/10.1016/j.jelechem.2017.11.023.

[2] a) V. Mirceski, R. Gulaboski, *J. Phys. Chem. B* **2006**, *110*, 2812-2820; b) B. Sefer, R. Gulaboski, V. Mirceski, *J. Solid State Electrochem.* **2012**, *16*, 2373-2381.
